# Author Correction: Pressure-induced charge amorphisation in BiNiO_3_

**DOI:** 10.1038/s41467-025-57973-6

**Published:** 2025-03-19

**Authors:** Wei-tin Chen, Takumi Nishikubo, Yuki Sakai, Hena Das, Masayuki Fukuda, Zhao Pan, Naoki Ishimatsu, Masaichiro Mizumaki, Naomi Kawamura, Saori I. Kawaguchi, Olga Smirnova, Mathew G. Tucker, Tetsu Watanuki, Akihiko Machida, Shigehiro Takajo, Yoshiya Uwatoko, Yuichi Shimakawa, Mikio Takano, Masaki Azuma, J. Paul Attfield

**Affiliations:** 1 Center for Condensed Matter Sciences (CCMS), National Taiwan University, Taipei, Taiwan, ROC; 2 Center of Atomic Initiative for New Materials (AI-Mat), National Taiwan University, Taipei, Taiwan, ROC; 3 Taiwan Consortium of Emergent Crystalline Materials, National Science and Technology Council, Taipei, Taiwan, ROC; 4 Kanagawa Institute of Industrial Science and Technology, Ebina, Kanagawa Japan; 5 Materials and Structures Laboratory, Institute of Integrated Research, Institute of Science Tokyo, Yokohama, Japan; 6 Graduate School of Advanced Science and Engineering, Hiroshima University, 1-3-1 Kagamiyama, Higashihiroshima, Hiroshima Japan; 7 Japan Synchrotron Radiation Research Institute, SPring-8, Sayo, Hyogo Japan; 8 Institute for Chemical Research, Kyoto University, Uji, Kyoto Japan; 9 ISIS Neutron and Muon Source, Rutherford Appleton Laboratory, Chilton, Didcot, UK; 10 Synchrotron Radiation Research Center, National Institutes for Quantum Science and Technology (QST), Sayo, Hyogo Japan; 11 Institute for Solid State Physics, University of Tokyo, Kashiwa, Chiba Japan; 12 Research Institute for Production Development, Sakyo-ku, Kyoto, Japan; 13 Research Center for Autonomous Systems Materialogy (ASMat), Institute of Integrated Research, Institute of Science Tokyo, Yokohama, Kanagawa Japan; 14 Centre for Science at Extreme Conditions, University of Edinburgh, Edinburgh, UK; 15 School of Chemistry, University of Edinburgh, Edinburgh, UK; 16 Present Address: Neutron Science and Technology Center, Comprehensive Research Organization for Science and Society, Tokai, Ibaraki Japan; 17 Present Address: Advanced Manufacturing Research Institute, National Institute of Advanced Industrial Science and Technology, Tsukuba, Ibaraki Japan; 18 Present Address: Beijing National Laboratory for Condensed Matter Physics, Institute of Physics, Chinese Academy of Sciences, Beijing, China; 19 Present Address: Geodynamics Research Center, Ehime University, Matsuyama, Ehime Japan; 20 Present Address: Faculty of Science, Kumamoto University, Kurokami, Kumamoto Japan; 21 Present Address: Neutron Scattering Division, Oak Ridge National Laboratory, Oak Ridge, TN USA

**Keywords:** Phase transitions and critical phenomena, Structure of solids and liquids, Ceramics

Correction to: *Nature Communications* 10.1038/s41467-025-57247-1, published online 5 March 2025

In this article, the author name Naomi Kawamura was incorrectly written as Nomi Kawamura. The original article has been corrected.

