# Supplementary material for: Pressure-induced charge amorphisation in BiNiO3
Source: Nat Commun. 2025 Mar 5;16:2128. doi: 10.1038/s41467-025-57247-1 (PMC11882806; doi:10.1038/s41467-025-57247-1)
Supplement: Supplementary file 1 — Supplementary Information [file 41467_2025_57247_MOESM1_ESM.pdf]

## Supplementary Information for

# Pressure-Induced Charge Amorphisation in $BiNiO_3$

Wei-tin Chen[1,2,3], Takumi Nishikubo[4,5], Yuki Sakai[4,5,16], Hena Das[4,5], Masayuki Fukuda[5,17], Zhao Pan[5,18], Naoki Ishimatsu[6.19], Masaichiro Mizumaki[7,20], Nomi Kawamura[7], Saori I. Kawaguchi[7], Olga Smirnova[8], Mathew G. Tucker[9,21], Tetsu Watanuki[10], Akihiko Machida[10], ShigehiroTakajo[11], Yoshiya Uwatoko[11,16], Yuichi Shimakawa[8], Mikio Takano[8,12], Masaki Azuma[13,5,4*], and J. Paul Attfield[14,15*]

1 Center for Condensed Matter Sciences (CCMS), National Taiwan University, Taipei 10617, Taiwan.

2 Center of Atomic Initiative for New Materials (AI-Mat), National Taiwan University, Taipei 10617, Taiwan.

3 Taiwan Consortium of Emergent Crystalline Materials, National Science and Technology Council, Taipei 10622 Taiwan

4 Kanagawa Institute of Industrial Science and Technology, 705-1 Shimoimaizumi, Ebina, Kanagawa 243-0435, Japan

5 Materials and Structures Laboratory, Institute of Integrated Research, Institute of Science Tokyo, Yokohama 226-8501, Japan

6 Graduate School of Advanced Science and Engineering, Hiroshima University 1-3-1 Kagamiyama, Higashihiroshima, Hiroshima 739-8526, Japan

7 Japan Synchrotron Radiation Research Institute, SPring-8, 1-1-1 Koto, Sayo, Hyogo 679-5198, Japan

8 Institute for Chemical Research, Kyoto University, Uji, Kyoto 611-0011, Japan.

9 ISIS Facility, Rutherford Appleton Laboratory, Chilton, Didcot OX11 0QX, United Kingdom.

10 Synchrotron Radiation Research Center, National Institutes for Quantum Science and Technology (QST), Sayo, Hyogo 679-5148, Japan

11 Institute for Solid State Physics, University of Tokyo, Kashiwa, Chiba 277-8581, Japan

12 Research Institute for Production Development, Sakyo-ku, Kyoto 606-0805, Japan

13 Research Center for Autonomous Systems Materialogy (ASMat), Institute of Integrated Research, Institute of Science Tokyo, Yokohama, Kanagawa, 226-8501, Japan

14 Centre for Science at Extreme Conditions, University of Edinburgh, Edinburgh, EH9 3FD, United Kingdom.

15 School of Chemistry, University of Edinburgh, Edinburgh, EH9 3FJ, United Kingdom.

16 Present address: Neutron Science and Technology Center, Comprehensive Research Organization for Science and Society, 162-1 Shirakata, Tokai, Ibaraki, 319-1106, Japan.

17 Present address: Advanced Manufacturing Research Institute, National Institute of Advanced Industrial Science and Technology, Tsukuba Central 5, 1-1-1 Higashi, Tsukuba, Ibaraki, 305-8565, Japan.

18 Present address: Beijing National Laboratory for Condensed Matter Physics, Institute of Physics, Chinese Academy of Sciences, Beijing 100190, China

19 Present address: Geodynamics Research Center, Ehime University, 2-5 Bunkyo, Matsuyama, Ehime, 790-8577, Japan.

20 Present address: Faculty of Science, Kumamoto University, Kurokami, Kumamoto, 860-8555, Japan.

21 Present address: Neutron Scattering Division, Oak Ridge National Laboratory, Oak Ridge, Tennessee 37831, United States.

* Corresponding authors: mazuma@msl.titech.ac.jp

* Corresponding authors: j.p.attfield@ed.ac.uk

Table S1. Rietveld refinement results for $BiNiO_3$ Phase-Id in space group *Pb*11 from NPD and SXRD. Lattice parameters, atomic coordination and refinement reliability $R_{wp}$ and *GOF* are listed.

| | NPD | | | | SXPD | | | |
|---|---|---|---|---|---|---|---|---|
| P (GPa) | 4.3 | | | | 4.3 | | | |
| T (K) | 100 | | | | 90 | | | |
| *a* (Å) | 5.2470(3) | | | | 5.2538(2) | | | |
| *b* (Å) | 5.5938(3) | | | | 5.6073(2) | | | |
| *c* (Å) | 7.6145(4) | | | | 7.6278(2) | | | |
| *α* (º) | 90.16(1) | | | | 90.148(2) | | | |
| V ($Å^3$) | 223.61(1) | | | | 224.71(1) | | | |
| $R_{wp}$ (%) | 2.59 | | | | 6.21 | | | |
| *GOF* | 1.08 | | | | 2.48 | | | |
| *Pb*11 [a] | x | y | z | $U_{iso}$ ($Å^2$) [b] | x | y | z | $U_{iso}$ ($Å^2$) [c] |
| Bi1 | 0.726(3) | 0.075(1) | 0.340(3) | 0.35(8) | 0.726(2) | 0.0711(6) | 0.3368(8) | 0.35 |
| Bi2 | 0.239(3) | 0.459(1) | 0.836(3) | 0.35 | 0.232(2) | 0.4614(5) | 0.848(1) | 0.35 |
| Ni1 | 0.251(3) | 0.025(3) | 0.088(2) | 0.05(4) | 0.24(2) | 0.01(2) | 0.09(1) | 0.05 |
| Ni2 | 0.255(3) | 0.019(3) | 0.584(2) | 0.05 | 0.25(2) | 0.01(2) | 0.58(1) | 0.05 |
| O1 | 0.420(4) | 0.346(3) | 0.135(2) | 0.01(4) | 0.45(3) | 0.35(2) | 0.13(2) | 0.01 |
| O2 | 0.939(4) | 0.216(3) | 1.016(3) | 0.01 | 0.88(3) | 0.29(2) | 1.00(2) | 0.01 |
| O3 | 0.082(4) | 0.704(4) | 0.648(2) | 0.01 | 0.07(3) | 0.60(3) | 0.70(2) | 0.01 |
| O4 | 0.533(3) | 0.795(3) | 0.508(3) | 0.01 | 0.55(3) | 0.87(3) | 0.58(2) | 0.01 |
| O5 | 0.875(2) | 0.442(2) | 0.332(4) | 0.01 | 0.77(2) | 0.50(2) | 0.34(3) | 0.01 |
| O6 | 0.381(2) | 0.041(2) | 0.838(4) | 0.01 | 0.42(2) | 0.07(3) | 0.87(3) | 0.01 |

[a] For the *Pb*11 (No. 7) model all atom positions are 2*a*, (*x*, *y*, *z*). [b] The $U_{iso}$ is constrained based on elements in the refinement. [c] The $U_{iso}$ from the NPD refinement results are adapted to the SXRD refinement.

Table S2. Selected bond distances and BVS results of Bi and Ni sites from NPD Rietveld refinement results of $BiNiO_3$ Phase-Id in space group *Pb*11.

| | | | |
|---|---|---|---|
| Bi1-O1 | 2.16(3) | Bi2-O1 | 2.55(3) |
| Bi1-O1 | 2.71(3) | Bi2-O1 | 3.61(3) |
| Bi1-O2 | 2.82(4) | Bi2-O2 | 2.49(3) |
| Bi1-O2 | 3.63(3) | Bi2-O2 | 2.19(3) |
| Bi1-O3 | 2.65(3) | Bi2-O3 | 2.63(3) |
| Bi1-O3 | 3.65(3) | Bi2-O3 | 2.15(3) |
| Bi1-O4 | 2.26(3) | Bi2-O4 | 2.92(3) |
| Bi1-O4 | 2.23(3) | Bi2-O4 | 3.49(3) |
| Bi1-O5 | 3.24(2) | Bi2-O6 | 3.26(2) |
| Bi1-O5 | 3.63(1) | Bi2-O6 | 2.46(1) |
| Bi1-O5 | 2.20(1) | Bi2-O6 | 2.05(2) |
| Bi1-O5 | 2.22(2) | Bi2-O6 | 3.34(1) |
| BVS | 4.1 | BVS | 4.0 |

| | | | |
|---|---|---|---|
| Ni1-O1 | 2.03(3) | Ni2-O3 | 2.04(3) |
| Ni1-O1 | 2.03(3) | Ni2-O3 | 2.11(3) |
| Ni1-O2 | 2.07(3) | Ni2-O4 | 2.01(3) |
| Ni1-O2 | 2.03(3) | Ni2-O4 | 1.99(3) |
| Ni1-O5 | 2.03(4) | Ni2-O5 | 2.08(4) |
| Ni1-O6 | 2.02(4) | Ni2-O6 | 2.05(4) |
| BVS | 2.2 | BVS | 2.3 |

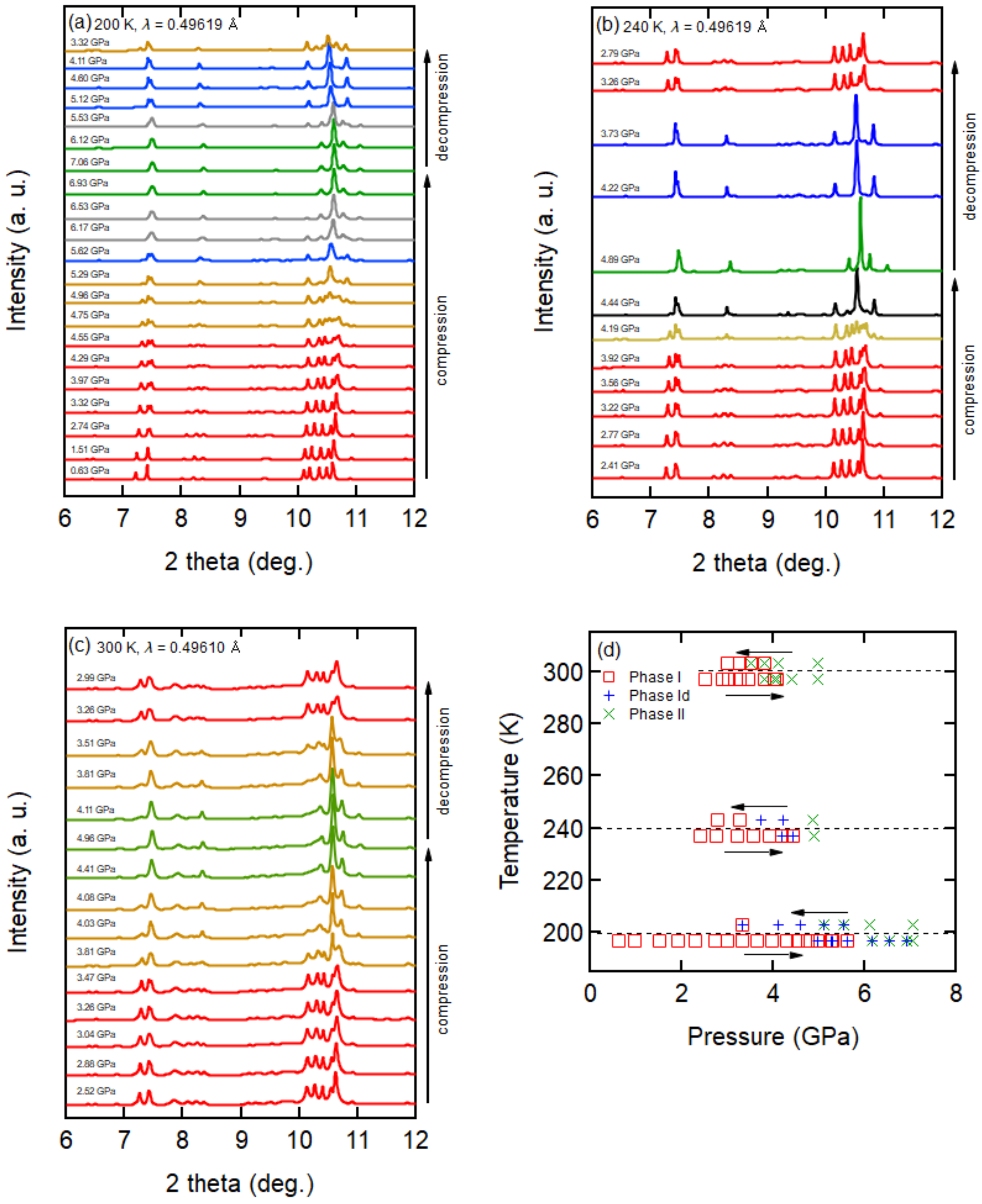


Fig. S1 Pressure-dependent synchrotron X-ray diffraction patterns of $BiNiO_3$ at (a) 200 and (b) 240 K, and (c) 300 K on compression and decompression, showing the phase transition from Phases-I to Id to II and *vice versa*. Red, blue and green lines indicate Phase-I, Id and II, respectively. Brown and grey lines denote the coexistence of two phases. Black line indicates the coexistence of three phases. (d) The pressure and temperature conditions of phase transitions and hysteresis are represented.

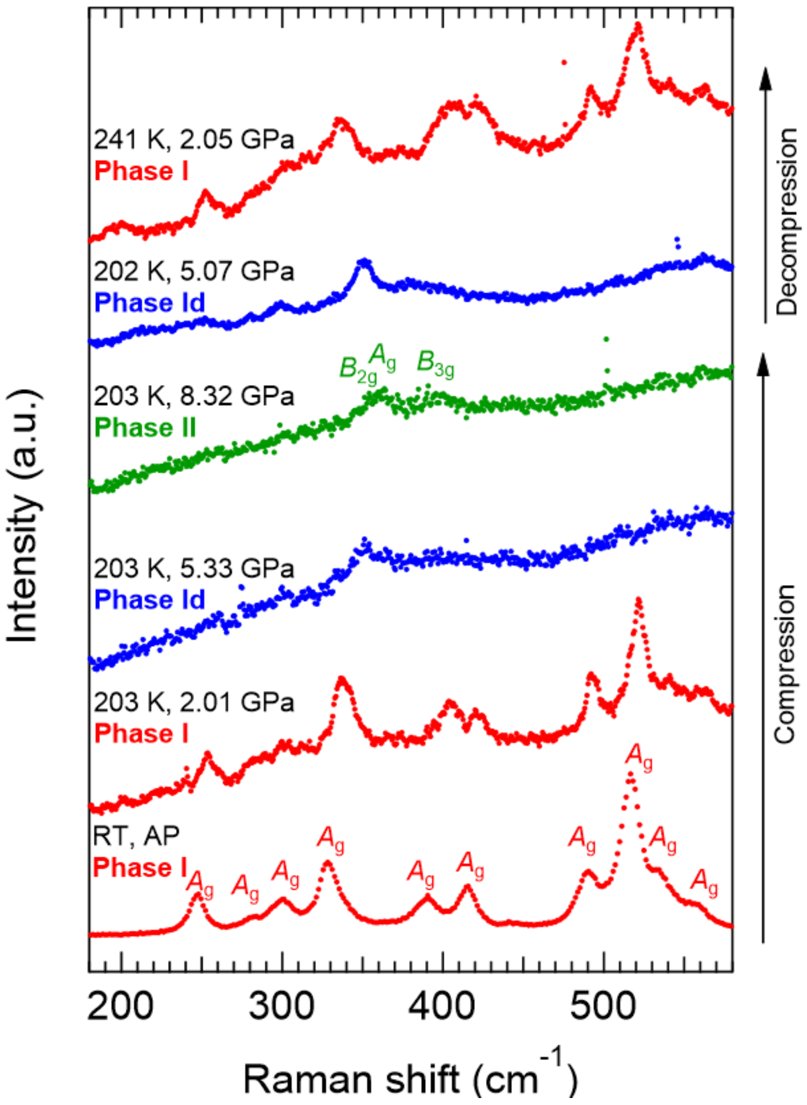


Fig. S2 Raman spectra of $BiNiO_3$ Phases-I, Id and II measured at high-pressure low-temperature conditions.

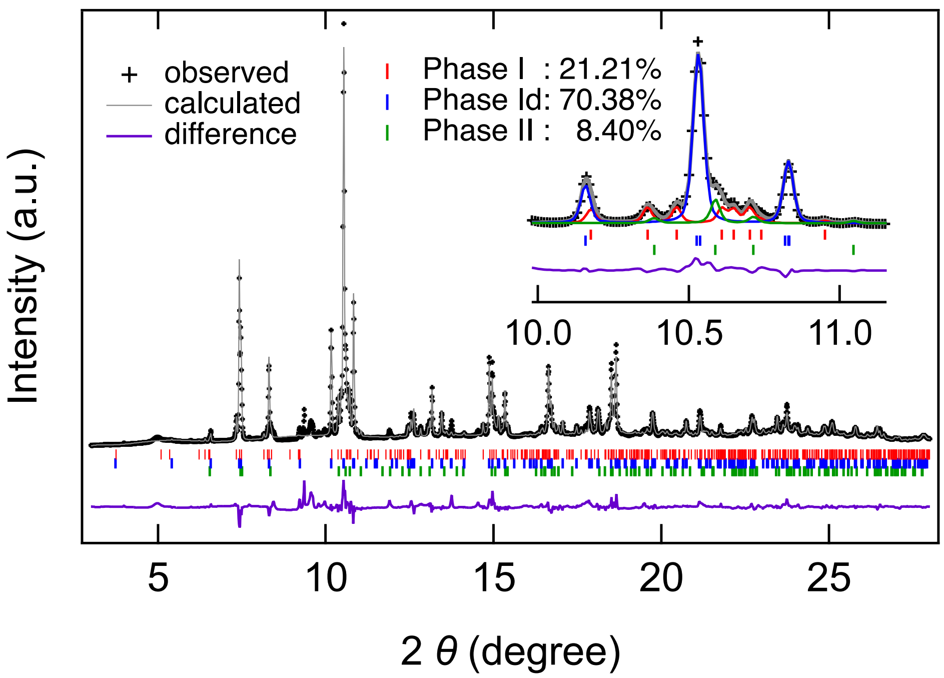


Fig. S3 Rietveld refinement result of $BiNiO_3$ at triple point (240 K and 4.44 GPa).

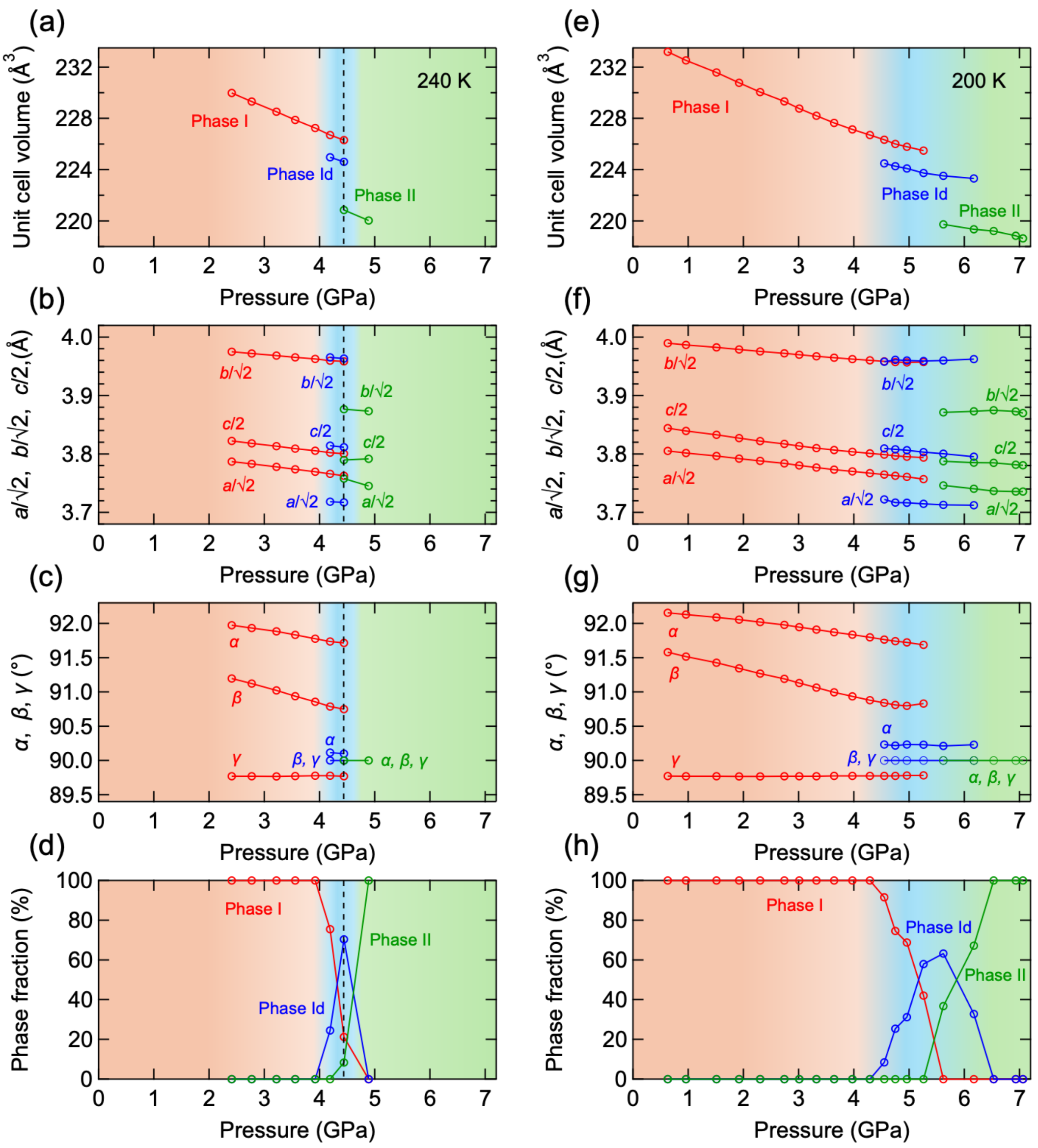


Fig. S4 Pressure dependence of unit cell volume (a, e), lattice constants (b, c, f, g) and phase fraction (d, h) at 240 K (a-d) and 200 K (e-h) obtained from the Rietveld refinements of SXRD data.

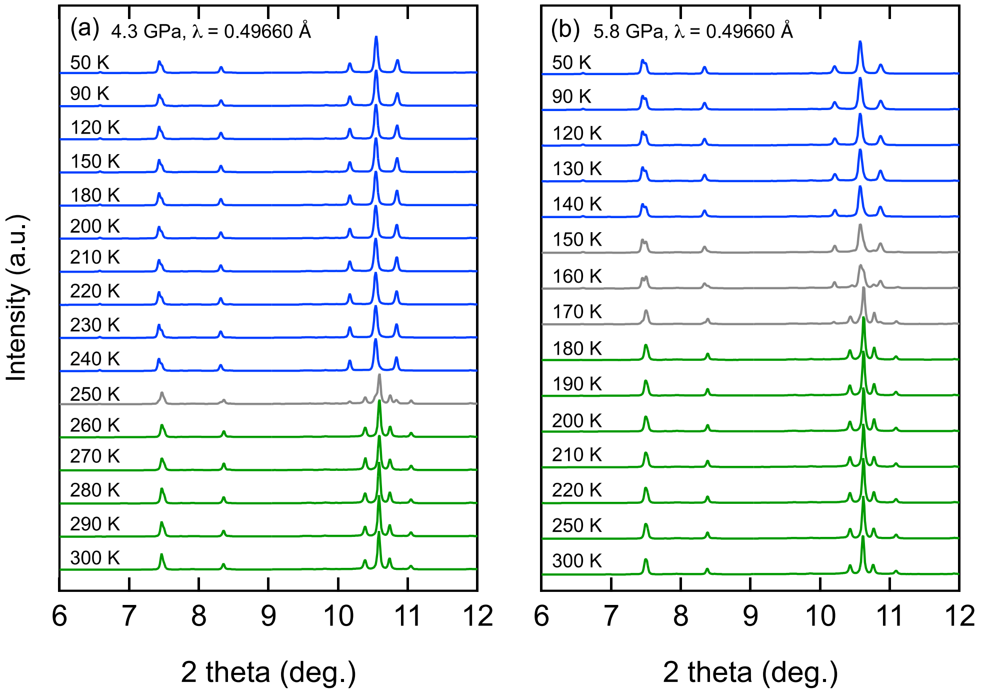


Fig. S5 Temperature-dependent synchrotron X-ray diffraction patterns of $BiNiO_3$ at (a) 4.3 and (b) 5.8 GPa on cooling. Green, blue and grey lines indicate Phase-II, Id and the coexistence of both phases, respectively.
